# Supplementary material for: Metabolic shift underlies recovery in reversible infantile respiratory chain deficiency
Source: EMBO J. 2020 Oct 31;39(23):e105364. doi: 10.15252/embj.2020105364 (PMC7705457; doi:10.15252/embj.2020105364)
Supplement: Supplementary file 1 — Expanded View Figures PDF [file EMBJ-39-e105364-s001.pdf]

Expanded View Figures

Figure EV1. Pedigrees, mtDNA haplotypes and variant counts in the 19 RIRCD families with homoplasmic m.14674T>C.

A Pedigrees and genetic variants. The * symbol indicates the individuals where DNA analysis was performed while circles represent female, square represents male and the filled-in square or circle represent individuals expressing mutations while deceased individuals were marked with a diagonal line.

B Exome mtDNA read depth and mtDNA haplogroups in the families homoplasmic for m.14674T>C.

C Variant Scatter/boxplot shows the significant difference between mean per group damaging allele counts (RIRCD affected vs unaffected vs 1,044 × 1000 Genomes control exomes) in the genes *EARS2, TRMU, QRSL1, GOT2, GLS, MSS51* and m.14674T>C. The counts are from individual patients and controls thus one point on the boxplot represents damaging allele counts for 1 person. The central band represents the median, the lower and upper hinges correspond to the first and third quartiles (25 and 75%) while the whiskers extend to the highest and lowest points within the data (1.5× the inter-quartile range).

 © 2020 The Authors

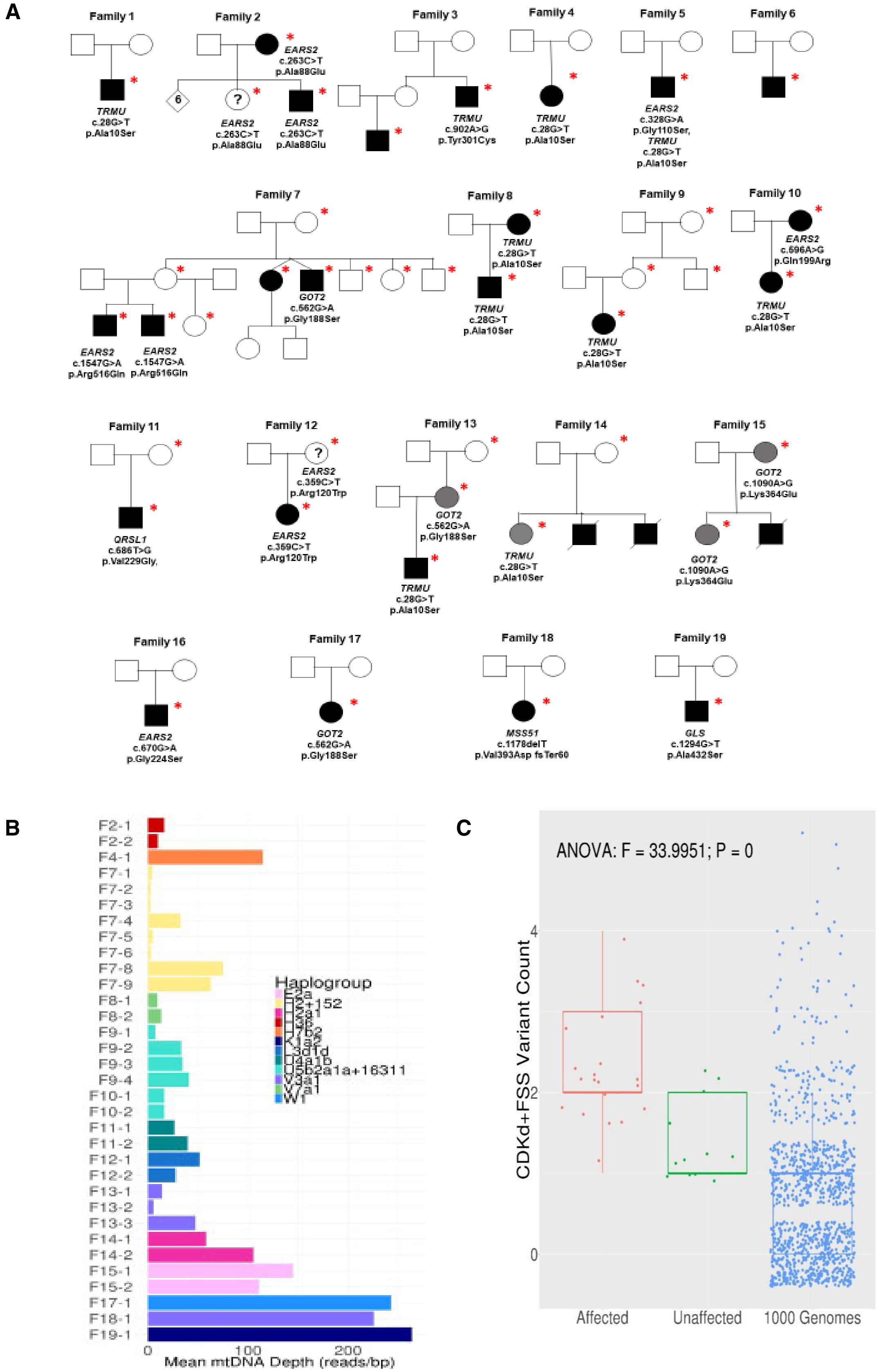
A
Family 1
TRMU
c.28G>T
p.Ala10Ser
Family 2
EARS2
c.263C>T
p.Ala88Glu
Family 3
TRMU
c.902A>G
p.Tyr301Cys
Family 4
TRMU
c.28G>T
p.Ala10Ser
Family 5
EARS2
c.328G>A
p.Gly110Ser,
TRMU
c.28G>T
p.Ala10Ser
Family 6
Family 7
EARS2
c.1547G>A
p.Arg516Gln
GOT2
c.562G>A
p.Gly188Ser
Family 8
TRMU
c.28G>T
p.Ala10Ser
Family 9
TRMU
c.28G>T
p.Ala10Ser
Family 10
EARS2
c.596A>G
p.Gln199Arg
TRMU
c.28G>T
p.Ala10Ser
Family 11
QRSL1
c.686T>G
p.Val229Gly,
Family 12
EARS2
c.359C>T
p.Arg120Trp
Family 13
GOT2
c.562G>A
p.Gly188Ser
TRMU
c.28G>T
p.Ala10Ser
Family 14
TRMU
c.28G>T
p.Ala10Ser
Family 15
GOT2
c.1090A>G
p.Lys364Glu
Family 16
EARS2
c.670G>A
p.Gly224Ser
Family 17
GOT2
c.562G>A
p.Gly188Ser
Family 18
MSS51
c.1178delT
p.Val393Asp fs Ter60
Family 19
GLS
c.1294G>T
p.Ala432Ser
B
F2-1
F2-2
F4-1
F7-1
F7-2
F7-3
F7-4
F7-5
F7-6
F7-8
F7-9
F8-1
F8-2
F9-1
F9-2
F9-3
F9-4
F10-1
F10-2
F11-1
F11-2
F12-1
F12-2
F13-1
F13-2
F13-3
F14-1
F14-2
F15-1
F15-2
F17-1
F18-1
F19-1
0
100
200
Mean mtDNA Depth (reads/bp)
Haplogroup
C
ANOVA: F = 33.9951; P = 0
CDKd+FSS Variant Count
0
2
4
Affected
Unaffected
1000 Genomes

Figure EV1.

Figure EV2. Summary of variants in our cohort.

A Mitochondrial haplogroups and digenic nuclear variants in affected RIRCD family members.

B Number of exome variants at each stage of filtering in total and for affected and unaffected individuals in RIRCD families with homoplasmic m.14674T>C.

 © 2020 The Authors

A

Family	Haplogroup	Relationship	Nuclear Risk Variants		
			Gene	Protein	Genotype
F1	n. d.	Proband	*TRMU*	p.Ala10Ser	0/1
F2	H36	Proband	*EARS2*	p.A88Glu	0/1
		Mother of F2/1	*EARS2*	p.Ala88Glu	0/1
F3	n. d.	Uncle of F3/2	*TRMU*	p.Tyr301Cy	0/1
F4	H7b2	Proband	*TRMU*	p.Ala10Ser	0/1
F5	V7a1	Proband	*EARS2*	p.Gly110Ser*	0/1
			TRMU	p.Ala10Ser	0/1
F7	H+152	Sibling of F7/2	*EARS2*	p.Arg516Gln*	0/1
		Sibling of F7/1	*EARS2*	p.Arg516Gln*	0/1
		Uncle of F7/1-2	*GOT2*	p.Gly188Ser	0/1
F8	U5b2a1a+16311	Proband	*TRMU*	p.Ala10Ser	0/1
F9	U5b2a1a+16311	Proband	*TRMU*	p.Ala10Ser	0/1
F10	U5b2a1a+16311	Proband	*TRMU*	p.Ala10Ser	0/1
		Mother of F8/1	*EARS2*	p.Gln199Arg	0/1
F11	U4a1b	Proband	*QRSL1*	p.Val229Gly	0/1
F12	L3d1d	Proband	*EARS2*	p.Arg120Trp	0/1
F13	V3a1	Proband	*TRMU*	p.Ala10Ser	0/1
		Mother of F13/1	*GOT2*	p.Gly188Ser	0/1
F14	H2a1	Sister of F14/1	*TRMU*	p.Ala10Ser	0/1
F15	E2a	Sister of F15/1	*GOT2*	p.Lys364Glu	0/1
		Mother of F15/1	*GOT2*	p.Lys364Glu	0/1
F17	W1	Proband	*GOT2*	p.Gly188Ser	0/1
F18	V3a1	Proband	*MSS51*	p.Val393AspfsTer60	0/1
F19	K1a2	Proband	*GLS*	p.Ala432Ser	0/1

B

Type of Variant	Total Number of Variants	Affected Only* (n=18)	Unaffected Only* (n=12)
Total Variants	4,907,159	1,847,621	1,106,024
Total Protein Altering	42,473	14,885	5,725
Conserved (2+)	15,775	6,051	2,344
Predicted Damaging (5+)	13,816	5,458	2,163
Conserved (2+), Damaging (5+)	10,991	4,340	1,705
Conserved (2+), Damaging (5+), Known Domain	5,013	2,022	779
**Conserved, Damaging, Known Domain, Splicing, Stop-Loss/Gain, Frameshift**	**6,998**	**2,698**	**1,067**

Figure EV2.

Figure EV3. Functional characterisation of the p.Arg23Trp variant in *PDE12*.

A Western blot of total cell lysate of *PDE12* knockout cells expressing WT *PDE12*, p.Glu351Ala (catalytic mutant), Δ16 (coding for *PDE12* lacking 16 first aa), Δ23 mutants (coding for *PDE12* lacking 23 first aa) and p.Arg23Trp *PDE12* cDNA. B-actin was used as loading control. Our previous study (Pearce *et al*, 2017) showed that the steady-state levels of OXPHOS complexes are affected in the PDE12 KO as compared to the WT control cells.

B Radioactive MPAT assay for 16S mt-rRNA extracted from HEK293T (WT), *PDE12* knockout cells (KO) and for *PDE12* KO cells expressing WT *PDE12*, p.Glu351Ala, Δ16, Δ23 mutants and p.Arg23Trp *PDE12* cDNA for 24 hr. Experiments and cell lines as described in (Pearce *et al*, 2017).

C Immunofluorescence to confirm mitochondrial localisation of the p.Arg23Trp *PDE12* variant in human cells. TOM20, translocator of the outer mitochondrial membrane 20. Scale bar = 20 μm.

Source data are available online for this figure.

 © 2020 The Authors

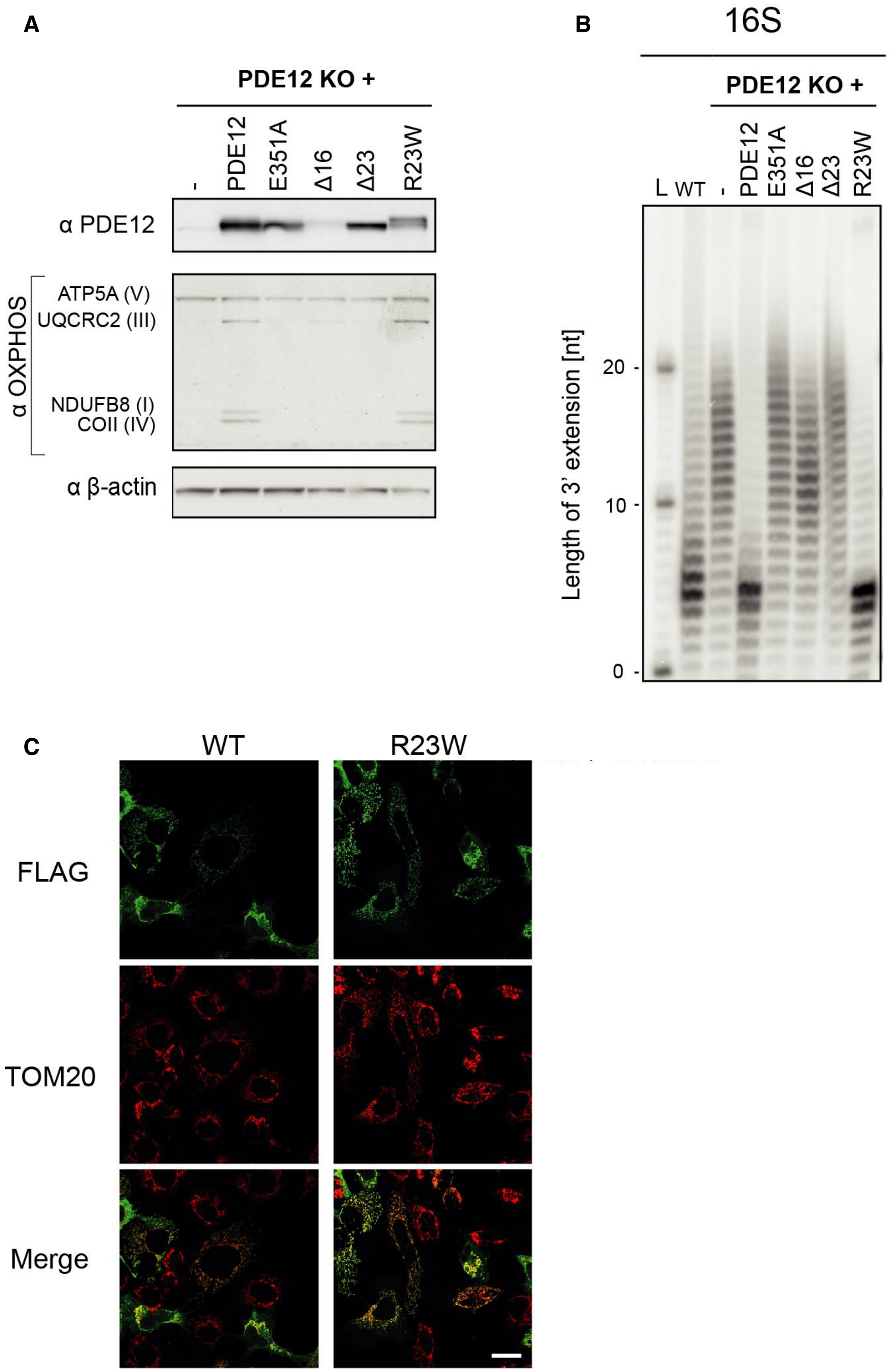
A
PDE12 KO +
-
PDE12
E351A
Δ16
Δ23
R23W
α PDE12
α OXPHOS
ATP5A (V)
UQCRC2 (III)
NDUFB8 (I)
COII (IV)
α β-actin
B
16S
PDE12 KO +
L
WT
-
PDE12
E351A
Δ16
Δ23
R23W
Length of 3' extension [nt]
20
10
0
C
WT
R23W
FLAG
TOM20
Merge

Figure EV3.

Figure EV4. Heat map of the top 100 transcripts altered in RNA-seq data resulted from muscle biopsies of RIRCD compared to control muscle resulted from DESeq2 analysis.
Gene expression is showed in normalized $\log_2$ counts. Differentially expressed genes were selected based on the $\log_2$ counts and *P*adj < 0.05.

 © 2020 The Authors

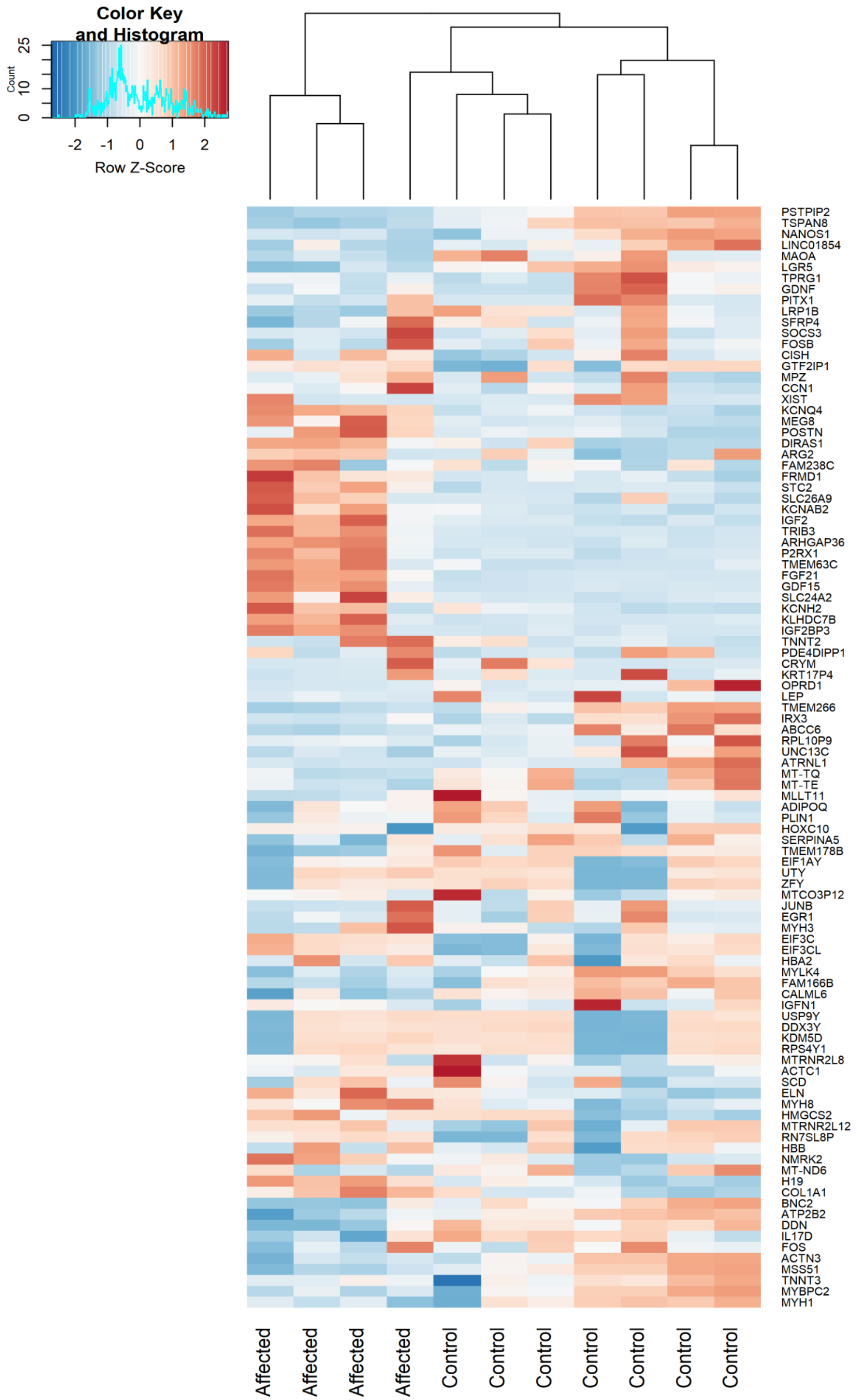
Color Key
and Histogram
Count
0
10
25
-2
-1
0
1
2
Row Z-Score
PSTPIP2
TSPAN8
NANOS1
LINC01854
MAOA
LGR5
TPRG1
GDNF
PITX1
LRP1B
SFRP4
SOCS3
FOSB
CISH
GTF2IP1
MPZ
CCN1
XIST
KCNQ4
MEG8
POSTN
DIRAS1
ARG2
FAM238C
FRMD1
STC2
SLC26A9
KCNAB2
IGF2
TRIB3
ARHGAP36
P2RX1
TMEM63C
FGF21
GDF15
SLC24A2
KCNH2
KLHDC7B
IGF2BP3
TNNT2
PDE4DIPP1
CRYM
KRT17P4
OPRD1
LEP
TMEM266
IRX3
ABCC6
RPL10P9
UNC13C
ATRNL1
MT-TQ
MT-TE
MLLT11
ADIPOQ
PLIN1
HOXC10
SERPINA5
TMEM178B
EIF1AY
UTY
ZFY
MTCO3P12
JUNB
EGR1
MYH3
EIF3C
EIF3CL
HBA2
MYLK4
FAM166B
CALML6
IGFN1
USP9Y
DDX3Y
KDM5D
RPS4Y1
MTRNR2L8
ACTC1
SCD
ELN
MYH8
HMGCS2
MTRNR2L12
RN7SL8P
HBB
NMRK2
MT-ND6
H19
COL1A1
BNC2
ATP2B2
DDN
IL17D
FOS
ACTN3
MSS51
TNNT3
MYBPC2
MYH1
Affected
Affected
Affected
Affected
Control
Control
Control
Control
Control
Control
Control

Figure EV4.

Figure EV5. Aminoacylation assay in fibroblasts.

Aminoacylation assay of mt-tRNA^{Glu} and mt-tRNA^{Gln} in fibroblasts carrying digenic mutations in mtDNA (m.14674T>C) and in *EARS2* (p.Arg516Gln; F7/1M, F7/2M), carrying only m.14674T>C (F7/5F, F7/7F) and healthy controls. The cells were cultures in low (low aa) and high amino acid (aa) concentrations. "dAc" indicated deacylated samples while "Ac" represents the aminoacylated tRNA. There is no relevant difference in the aminoacylation of fibroblasts of patients with digenic mutations, carriers of m.14674T>C and healthy controls.

 © 2020 The Authors

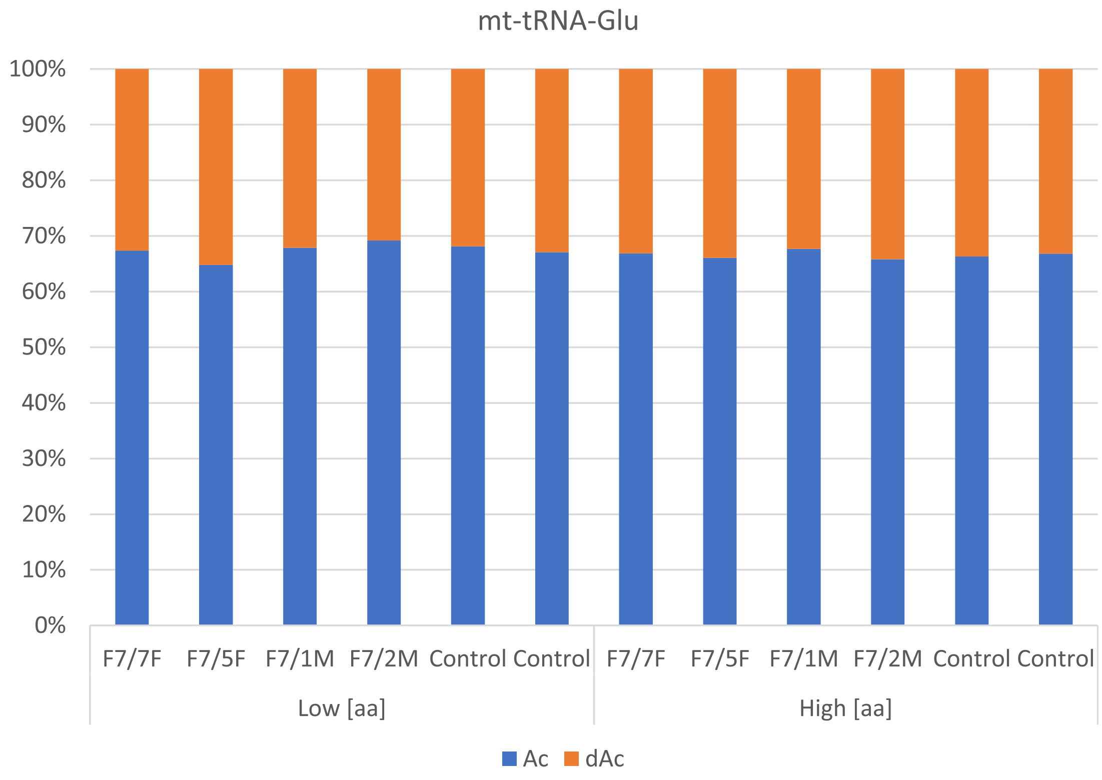
mt-tRNA-Glu
100%
90%
80%
70%
60%
50%
40%
30%
20%
10%
0%
F7/7F F7/5F F7/1M F7/2M Control Control
Low [aa]
F7/7F F7/5F F7/1M F7/2M Control Control
High [aa]
Ac dAc

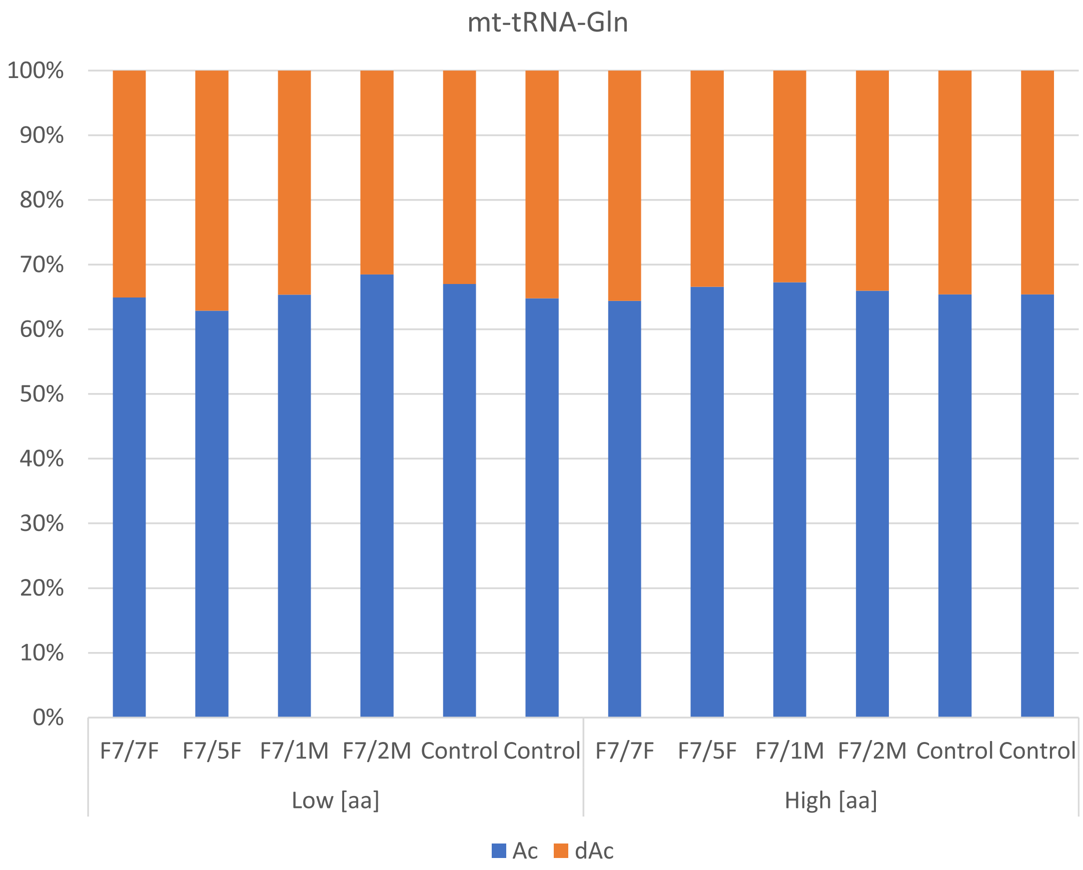
mt-tRNA-Gln
100%
90%
80%
70%
60%
50%
40%
30%
20%
10%
0%
F7/7F F7/5F F7/1M F7/2M Control Control
Low [aa]
F7/7F F7/5F F7/1M F7/2M Control Control
High [aa]
Ac dAc

Figure EV5.
