# Supplementary material for: Metabolic shift underlies recovery in reversible infantile respiratory chain deficiency
Source: EMBO J. 2020 Oct 31;39(23):e105364. doi: 10.15252/embj.2020105364 (PMC7705457; doi:10.15252/embj.2020105364)
Supplement: Supplementary file 2 — Table EV1 [file EMBJ-39-e105364-s002.docx]

**Table EV1.** Summary of clinical presentations and genotypes of the index patients and their affected and unaffected family members in this study, all carrying the homoplasmic m.14674T>C mt-tRNAGlu mutation.

| **Family**  **Patient** | **Family history** | **Age of onset** | **Infantile clinical presentation** | | | | **Age of**  **recovery** | **Residual symptoms** | **References**  **(ethnic origin)** | **Homoplasmic m.14674T>C**  **plus additional variants** | **Muscle RNAseq**  **proteomics** |
| --- | --- | --- | --- | --- | --- | --- | --- | --- | --- | --- | --- |
|  |  |  | **Muscle** | **Ventila-ted** | **Tube fed** | **Liver** |  |  |  |  |  |
| F1/1M | Index patient | birth | + |  |  |  | 4 m | 5 y healthy | Horvath 2009  (USA) | het ***TRMU*** c.28G>T;p.Ala10Ser |  |
| F2/1 M | Index patient | 15 days | + | + | + |  | 5 m | motor delay | Horvath 2009  (USA) | het ***EARS2*** c.263C>TA;p.Ala88Glu | RNAseq proteomics |
| F2/2 F | Mother of F2/1 | n. d. | + |  |  |  | n. d. | mild motor delay | Horvath 2009  (USA) | het ***EARS2*** c.263C>A;p.Ala88Glu | RNAseq |
| F3/1M | Maternal uncle of F3/2 | 1 m | + |  | + | + | 10-22 m | 22 y mild myopathy | Horvath 2009  (Sweden) | het ***TRMU*** c.902A>G; p.Tyr301Cys |  |
| F3/2M | Index patient | 1 m | + | + | + |  | 7-18 m | 7 y healthy | Horvath 2009  (Sweden) |  | RNAseq proteomics |
| F4/1F | Index patient | birth | + |  | + |  | 9-36 m | 17 y mild myopathy | Houshmand 1994 (Sweden) | het ***TRMU*** c.28G>T;p.Ala10Ser | RNAseq |
| F5/1M | Index patient | 2 m | + |  | + |  | 5-19 m | 7 y healthy | Houshmand 1994 (Sweden) | het ***TRMU*** c.28G>T; p.Ala10Ser  het***EARS2***c.328G>A;pGly110Ser | RNAseq proteomics |
| F6/1M | Index patient | 3 m | + |  |  |  | 12 m | healthy | This paper  (Italy) |  | RNAseq |
| F7/1M | Index patient | birth | + | + | + | + | 6-30 m | 19 y mild myopathy | Horvath 2009  (Germany) | het ***EARS2*** c.1547G>A; p.Arg516Gln |  |
| F7/2M | Sibling of F7/1 | 2.5 m | + | + | + |  | 5-20 m | 17 y mild myopathy | Horvath 2009  (Germany) | het ***EARS2*** c.1547G>A; p.Arg516Gln |  |
| F7/3F | Maternal aunt of F7/1 | 1 m | + |  | + |  | 5-20 m | 34 y healthy | Horvath 2009  (Germany) |  |  |
| F7/4M | Maternal uncle of F7/1 | 1 m | + |  | + |  | 5-18 m | 34 y healthy | Horvath 2009  (Germany) | het ***GOT2*** c.562G>A;  p.Gly188Ser |  |
| F7/5F | Mother of F7/1,2 |  |  |  |  |  |  | 39 y healthy |  |  |  |
| F7/6F | Half-sister o F7/1,2 |  |  |  |  |  |  | 7 y healthy |  |  |  |
| F7/7F | Daughter of F7/3 |  |  |  |  |  |  | 7 y healthy |  |  |  |
| F7/8M | Brother of F7/5 |  |  |  |  |  |  | 34 y healthy |  |  |  |
| F7/9F | Sister of F7/5 |  |  |  |  |  |  | 37 y healthy |  |  |  |
| F7/10m | Son of F7/3 |  |  |  |  |  |  | 9 y healthy |  |  |  |
| F8/1M | Index patient | birth | + |  |  |  | 5 m | healthy | This paper  (Sweden) | het ***TRMU*** c.28G>T;p.Ala10Ser |  |
| F8/2F | Mother of F8/1 | n. d. |  |  |  |  |  | n. d. |  | het ***TRMU*** c.28G>T;p.Ala10Ser |  |
| F9/1F | Index patient | 2 m | + | + | + |  | 5 m | 5 y healthy | This paper (Estonia) | het ***TRMU*** c.28G>T;p.Ala10Ser |  |
| F9/2F | Mother of F91 |  |  |  |  |  |  | 30 y healthy |  |  |  |
| F9/3M | Uncle of F9/1 |  |  |  |  |  |  | 26 y healthy |  |  |  |
| F9/4F | Grandmother of F9/1 |  |  |  |  |  |  | 54 y healthy |  |  |  |
| F10/1F | Index patient | 1 m | + | + | + |  | 4-22 m | 14 y mild myopathy | Joost 2012 (Estonia) | het ***TRMU*** c.28G>T;p.Ala10Ser |  |
| F10/2F | Mother of F10/1 | first year | + |  |  |  | 1-2 y | mild myopathy | This paper (Estonia) | het ***EARS2*** c.596A>G; p.Gln199Arg |  |
| F10/3F | Grandmother of F10/1 |  |  |  |  |  |  | healthy |  |  |  |
| F11/1M | Index patient | 7 w | + |  | + |  | 6-20 m | 13 y mild myopa-thy, FGF21: 183 pg/ml (< 170) | This paper (Brazil) | het ***QRSL1*** c.686T>G;  p.(Val229Gly) |  |
| F11/2F | Mother of F11/1 |  |  |  |  |  |  | healthy |  |  |  |
| F12/1F | Index patient | 1 m | + | + | + |  | 1-2 y | 8 y ptosis, mild myopathy, FGF21: 204 pg/ml (< 240) | This paper (Brazil) | het ***EARS2*** c.358C>T; p.Arg120Trp |  |
| F12/2F | Mother of F12/1 | n. d. |  |  |  |  |  | healthy |  | het ***EARS2*** p.Arg120Trp |  |
| F13/1M | Index patient | birth | + | + | + |  | 6-12 m | Pearson syndrome, mild myopathy | This paper (UK) | het ***TRMU*** c.28G>T;p.Ala10Ser |  |
| F13/2F | Mother of F13/1 | n. d. | + | + | + |  | 6-12 m | mild myopathy | This paper (UK) | het ***GOT2*** c.562G>A;  p.Gly188Ser |  |
| F13/3F | Grandmother of F13/1 |  |  |  |  |  |  | healthy |  |  |  |
| F14/1F | Sister of index patient | n. d. |  |  |  |  |  | mild myopathy | Schara et al 2015 (Germany) | het ***TRMU*** c.28G>T;p.Ala10Ser |  |
| F14/2F | Mother of index patient |  |  |  |  |  |  | healthy |  |  |  |
| F15/1F | Sister of index patient | n. d. |  |  |  |  |  | 10 y mild myopathy | This paper (Canada) | het ***GOT2*** c.1090A>G;  p.(Lys364Glu) |  |
| F15/2F | Mother of F15/1 | n. d. |  |  |  |  |  | mild myopathy | This paper (Canada) | het ***GOT2*** c.1090A>G;  p.(Lys364Glu) |  |
| F16/1F | Index patient | 6 w | + | + | + |  | 6-15 m | 20 months, mild myopathy | Horvath et al., 2009 (US) | het ***EARS2*** c.670G>A;p.Gly224Ser |  |
| F17/1M | Index patient | 10 w | + | + | + |  | 4 m | mild myopathy | P3 Uusimaa et al 2011 (UK) | het ***GOT2*** c.562G>A;  p.Gly188Ser |  |
| F18/1F | Index patient, sister’s child also affected | 6 w | + |  | + |  | 9 m | pain, mild myopathy | P1 Uusimaa et al 2011 (UK) | het ***MSS51*** c.1178delT;  p.(Val393AspfsTer60) |  |
| F19/1F | Index patient | 2 w | + |  | + | + | 14-18 w | mild myopathy | P4 Uusimaa et al 2011 (Italy) | het ***GLS*** c.1294G>T;  p.(Ala432Ser), |  |
