# Supplementary material for: Metabolic shift underlies recovery in reversible infantile respiratory chain deficiency
Source: EMBO J. 2020 Oct 31;39(23):e105364. doi: 10.15252/embj.2020105364 (PMC7705457; doi:10.15252/embj.2020105364)
Supplement: Supplementary file 3 — Table EV2 [file EMBJ-39-e105364-s003.docx]

**Table EV2**. Details of digenic nuclear variants in affected RIRCD patients homoplasmic for m.14674T>C.

| Gene | Variant | Patient/Family | ExAc | gnomAD | ExAc homozygous | CADD score | Protein function |
| --- | --- | --- | --- | --- | --- | --- | --- |
| *EARS2* | p.Arg120Trp | 2/1 | 6.57x10^-5^ | 2.79x10^-5^ | 0 | 29.4 | tRNA synthetase of mt-tRNA^Glu^ |
|  | p.Gln199Arg | 1/1 | 1.66x10^-5^ | 1.4x10^-5^ | 0 | 8.1 |  |
|  | p.Ala88Glu | 2/1 | 1.66x10^-3^ | 1.15x10^-3^ | 0 | 23.6 |  |
|  | p.Gly110Ser* | 1/1 | 3.66x10^-4^ | 2.09x10^-4^ | 0 | 34 |  |
|  | p.Gly224Ser* | 1/1 | 1.20x10^-3^ | 3.38x10^-3^ | 4 | 28.3 |  |
|  | p.Arg516Gln* | 2/1 | 3.31x10^-5^ | 9.07x10^-5^ | 0 | 34 |  |
| *TRMU* | p.Ala10Ser | 9/8 | 0.197 | 9.69x10^-2^ | 303 | 29.6 | thiomodification of mt-tRNA^Glu^ |
|  | p.Tyr301Cys | 1/1 | 6.7x10^-4^ | 4.17x10^-4^ | 0 | 25.5 |  |
| *GOT2* | p.Gly188Ser | 3/3 | 0.064 | 6.04x10^-2^ | 309 | 24.6 | mitochondrial glutamate-oxaloacetate transaminase |
|  | p.Lys364Glu | 2/2 | 7.25x10^-4^ | 7.42x10^-4^ | 0 | 21.9 |  |
| *MSS51* | p.Val393AspfsTer60 | 1/1 | 0.002 | 1.51x10^-3^ | 0 | -  frame shift | muscle specific *MTCOI* translational activator |
| *QRSL1* | p.Val229Gly | 1/1 | 0.001 | 1.29x10^-3^ | 0 | 25.8 | tRNA synthetase of mt-tRNA^Glu^ and mt-tRNA^Gln^ |
| *GLS* | p.Ala432Ser | 1/1 | 0.001 | 1.67x10^-3^ | 1 | 25.9 | glutaminase: converts glutamine to glutamate |
