# Supplementary figures and images for: Metabolic shift underlies recovery in reversible infantile respiratory chain deficiency
Source: EMBO J. 2020 Oct 31;39(23):e105364. doi: 10.15252/embj.2020105364 (PMC7705457; doi:10.15252/embj.2020105364)

Figure EV3 A

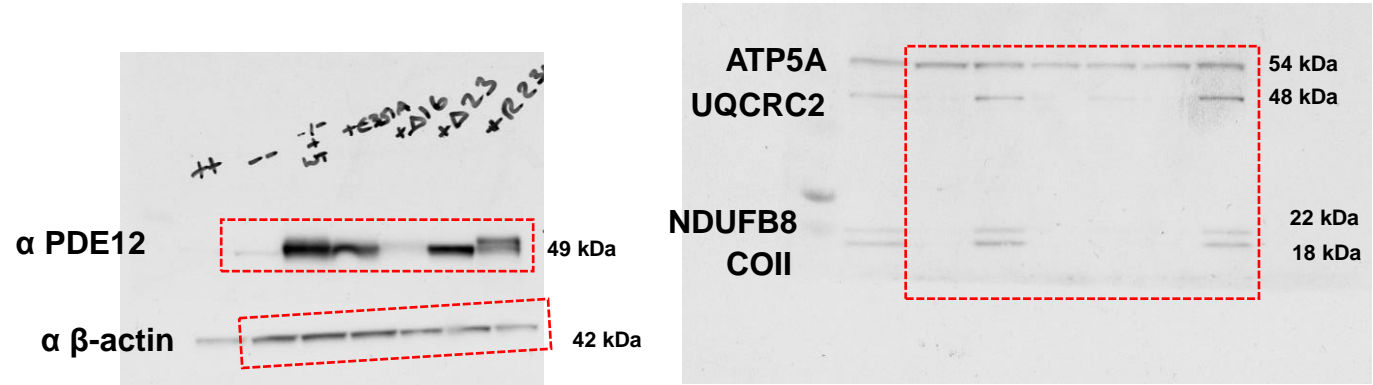

Supplement: Supplementary file 6 — Source Data for Expanded View [file EMBJ-39-e105364-s008.pdf]

Figure 3 A

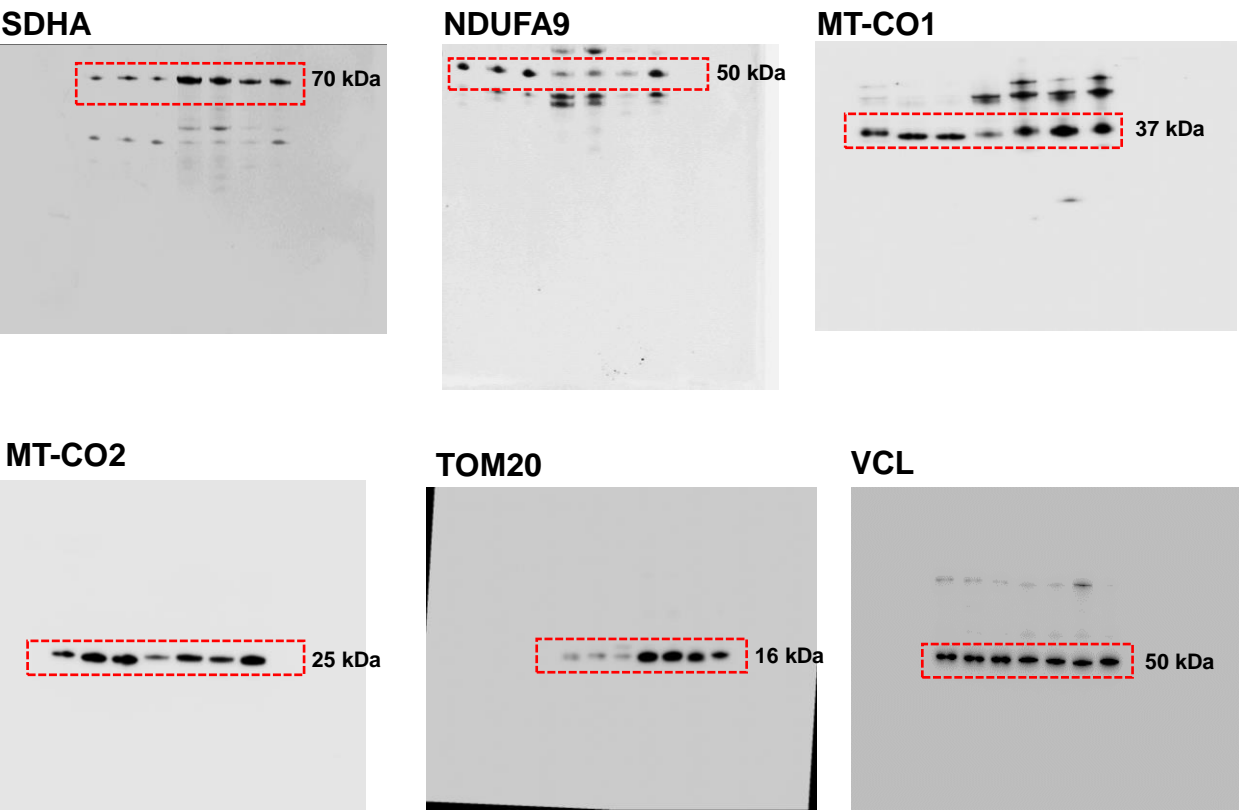

Figure 3 D

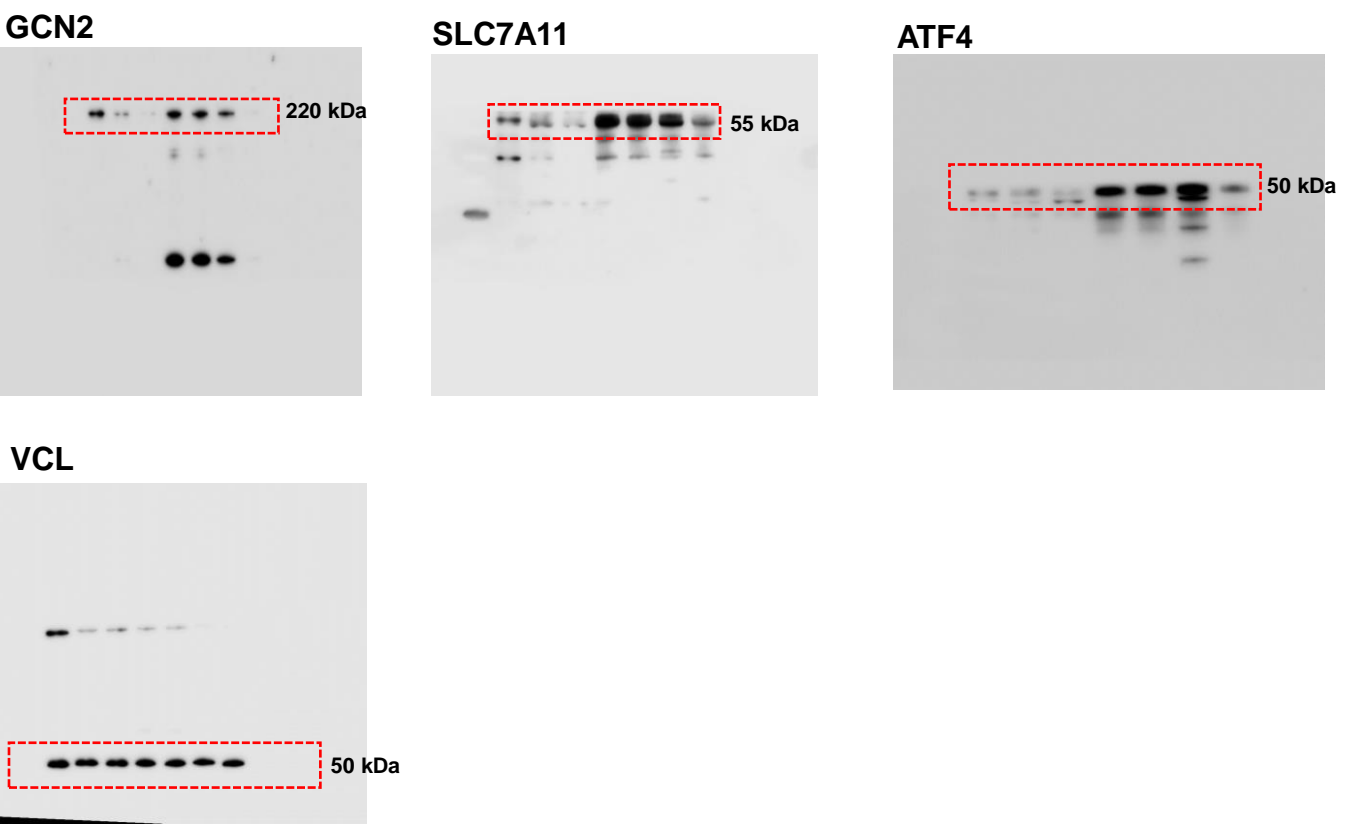

Supplement: Supplementary file 8 — Source Data for Figure 3 [file EMBJ-39-e105364-s006.pdf]

Figure 4 A

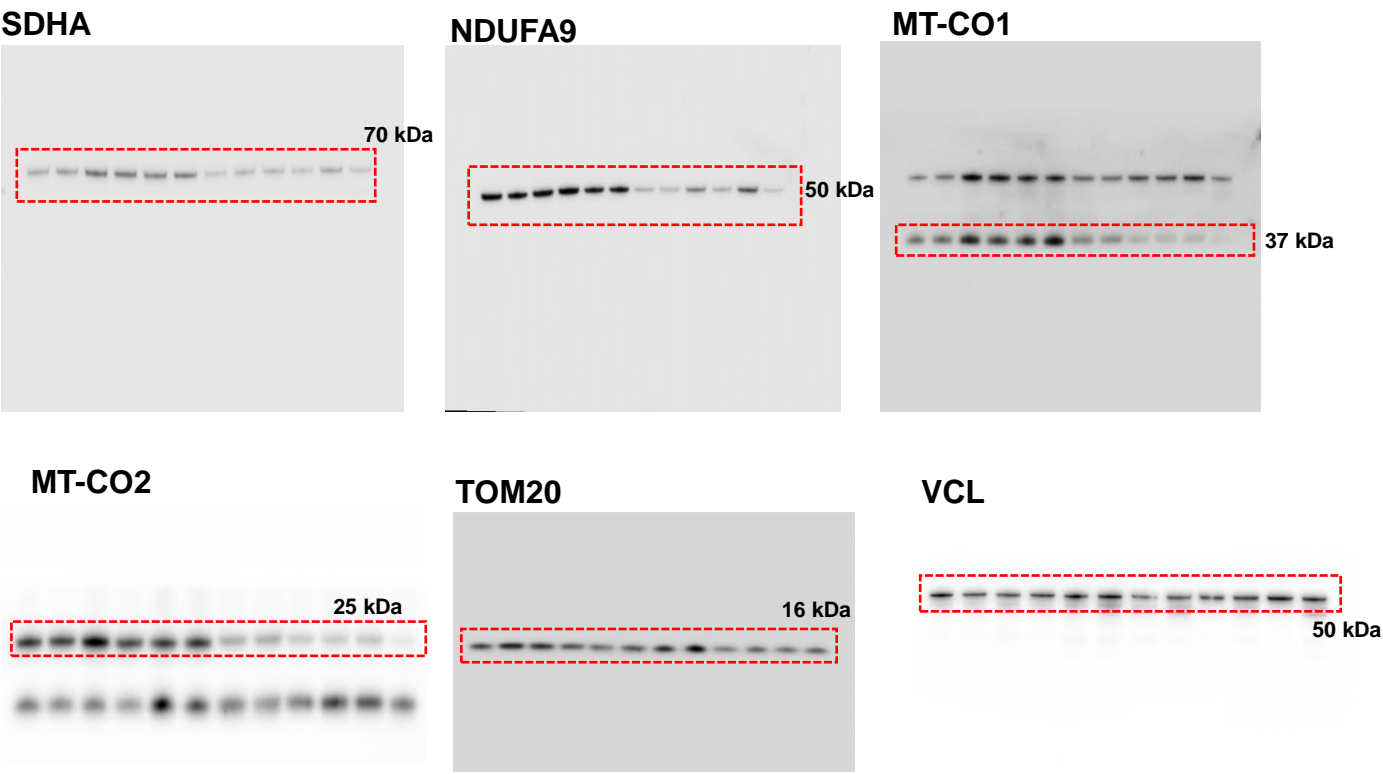

Figure 4 C

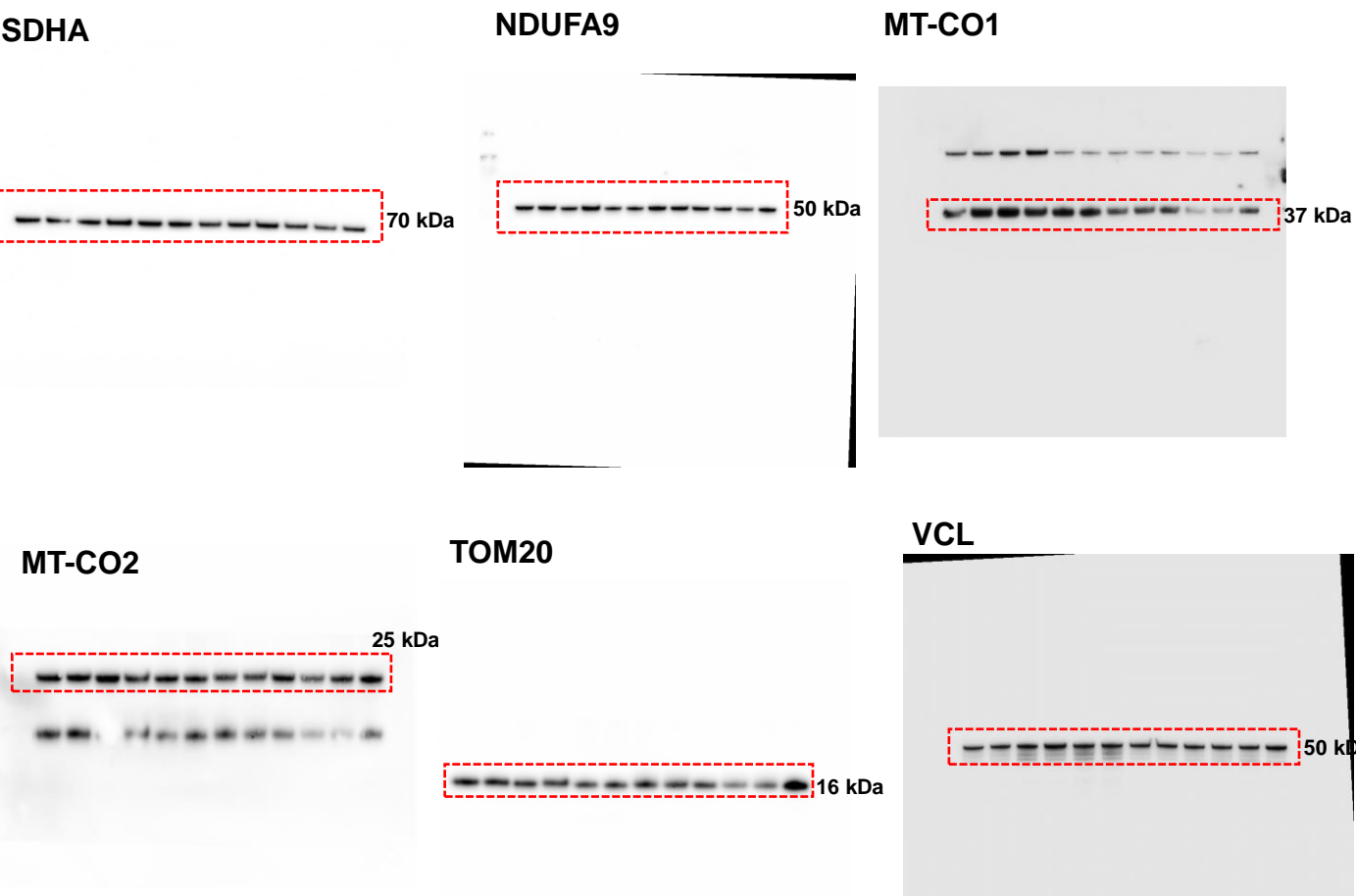

Supplement: Supplementary file 9 — Source Data for Figure 4 [file EMBJ-39-e105364-s007.pdf]
